# Ambulatory Sedation for Dental Procedures—Case of Cuenca, Ecuador

**DOI:** 10.3390/children9111618

**Published:** 2022-10-25

**Authors:** Eleonor María Velez-León, Karen Lozada Vargas, Katherine Cuenca-León, Cristina Acurio-Vargas, Adriana Zumba, Edisson-Mauricio Pacheco-Quito

**Affiliations:** 1 Academic Unit of Health and Wellness, Faculty of Dentistry, Catholic University of Cuenca, Cuenca 010105, Ecuador; 2 Research Group: Innovation and Pharmaceutical Development in Dentistry Research Group, Faculty of Dentistry, Head of Research and Innovation, Catholic University of Cuenca, Cuenca 010105, Ecuador; 3 SEDARTE, Cuenca 010105, Ecuador

**Keywords:** sedation, children, dentistry, dental procedure, drug

## Abstract

In recent years, the dental treatment of children under sedation and/or general anesthesia on an outpatient basis has been developed as a behavioral management model in pediatric dentistry. The objective of this study was to establish the percentage of pediatric patients who required deep sedation on an outpatient basis in dental offices in the city of Cuenca, Ecuador. An observational study was conducted with a sample of 450 records of school- and preschool-age patients, where the variables were type and time of treatment, age, and sex. Statistical data were analyzed using the statistical program SPSS V.27 (IBM, Armonk, NY, USA). The highest percentage of children who received sedation were of preschool age. In general, there were three types of procedures per session, the most frequent being restorations (67%), followed by pulp treatment (49.8%) and, less frequently, minor surgery. The need for sedation for dental procedures is high in preschool patients, and ambulatory sedation has contributed to meeting this need. However, a regulation for its use is required at a national level.

## 1. Introduction

Classically, dental care requiring sedation or general anesthesia has been provided in surgical or hospital settings [1]. However, during the last few decades in various countries, there has been an increasing trend of offering sedation or general anesthesia services in the dental office for the purposes of pain relief and anxiety control [2,3].

In pediatric dentistry, minimal pharmacological or non-pharmacological interventions are often not sufficient to achieve adequate comprehensive care, since factors such as the extensive treatment needs of the child, acute situational anxiety, age, limited cognitive functioning, long intervention times, physical disability, or medical conditions require deep sedation or general anesthesia to develop dental treatment safely [4,5,6,7,8]. In relation to adults, children have constantly changing anatomical, pharmacokinetic, and psychological differences; therefore, sedation aims to maintain safety, eliminate pain, reduce anxiety, and control behavior, allowing the planned intervention to be carried out [9,10].

Opinions on what constitutes sedation differ in the medical field, and it is essential to differentiate sedation from general anesthesia. Unfortunately, many sedative agents can also act as general anesthetics, and the difference in the dose required for a sedated patient and an anesthetized patient can be very small and highly variable among patients [11,12].

The management of sedation in dental offices requires that these procedures be performed under conditions of safety, efficacy, and under the supervision of qualified professionals [13], and that they comply with a protocol for diagnosis, evaluation, preparation, implementation, follow-up, and recovery from sedation [3], thus facilitating the performance of dental treatment and providing a positive patient experience [2].

Authorized bodies in the field such as the American Academy of Pediatric Dentistry (AAPD) [14] support the use of deep sedation or general anesthesia in the dental office, as long as it is administered by qualified personnel; among the benefits cited are early access to dental care, ease and efficiency in scheduling clinical intervention, reduction of administrative procedures, lower costs compared to surgical or hospital centers, decreased patient movement, and optimizing the quality of care [15]. It is necessary to point out that the AAPD suggests that these sedation or general anesthesia procedures be performed only if the patient’s orofacial risk is high; otherwise, it is recommended to use conventional procedures, since complications during the performance of the procedure are common and well documented [9,14]. In addition, the US Food and Drug Administration (FDA) in December 2016 announced that exposure to certain sedatives and general anesthetics may affect brain development in children under 3 years of age, especially in procedures lasting more than 3 h [16], a situation that has been discussed for several years, without definitive conclusions [17]. This official warning was recently included in the manufacturers’ package inserts of 11 commonly used drugs and sedatives, such as volatile inhalation agents including halothane, desflurane, isoflurane, and sevoflurane; intravenous anesthetics including propofol, methohexital, and etomidate; ketamine; and sedative hypnotics including lorazepam (injection), midazolam (injection and syrup), and pentobarbital. It is unclear at this time how this will affect the practice of clinical dental anesthesia in the near future [18,19,20].

In Latin America, there is little information on the use of sedation or general anesthesia in dental offices. In the case of Ecuador, ambulatory anesthesia became a necessity during the time of COVID-19 confinement, since access to a hospital environment to treat children’s, dental needs was practically impossible [21]. In this context, the dental care of preschool children under sedation in the hospital setting has been limited, so the outpatient sedation service has been promoted. In the case of Ecuador, there is no specific regulation that regularizes the sedation in the dental office, professionals have relied on international regulations, such as the regulation of Colombia, where it is mentioned that endovenous sedation procedures should be performed exclusively by anesthesiologists [22], since due to their competence, dentists could not perform this type of procedure. In addition, the policies proposed by the AAPD [14] for this type of procedure emphasize the importance of sedation providers being licensed, accredited, and certified in pediatric advanced life support (PALS). In addition, facilities must comply with all local, state, and federal laws, codes, and regulations pertaining to the provision of anesthesia services, controlled storage of medications, fire prevention, patient safety, and accommodations for the disabled [22,23].

Therefore, the need arises to develop this type of study, which consists of recording data on the need for sedation in dental offices by age group and type of treatment performed in the city of Cuenca, Ecuador.

## 2. Materials and Methods

The research was observational, cross-sectional, and documentary, with a sample of 450 cards belonging to children aged 1 to 12 years who received dental treatment for different etiologies, and in turn were attended by a group of professionals who offered sedation and general anesthesia service on an outpatient basis during the years 2019–2021.

The permission of the Bioethics Committee of the Catholic University of Cuenca, Ecuador (CEISH) was not necessary because this was a documentary study involving anonymized and coded records that were in a database to which the researchers had access.

### 2.1. Inclusion Criteria

Treated files that passed a quality control review of 10% of their totality to avoid erroneous data and/or duplicates.

### 2.2. Exclusion Criteria

Files that did not meet the quality criteria, were incorrectly filled out, or were duplicates.

### 2.3. Variables

In this study, the variables analyzed were age, sex, time of dental treatment, and type of dental treatment (restorations, pulp treatment, exodontia, minor surgery).

### 2.4. Statistical Analysis

The research is presented using frequency measures and the mode is mainly reported. Associations were made using the Chi-square statistic with a significance of *p* < 0.05. Data processing was performed in the SPSS V27 statistical program.

### 2.5. Procedure

Conscious sedation was performed by administering sevoflurane, an anesthetic gas that is mixed with oxygen to induce sleep in the first instance. Remifentanil and propofol were administered intravenously at a minimal dose compared to the dose of general anesthesia; for the post-surgical process and depending on the need of each patient, ketorolac and dexamethasone were used. All doses were calculated according to the patient’s weight.

## 3. Results

### 3.1. Participants and Sedation Conditions

A total of 448 children between 1 year 1 month and 12 years 9 months participated in the study. The population was divided into three age groups, the first one with ages ranging from 1 to 5 years 11 months who presented mainly primary dentition; the second group with children between 6 and 9 years 11 months with mixed dentition and a predominance of primary dentition; and the last group with children between 10 and 12 years 9 months with mixed dentition, with a predominance of permanent teeth. Sedation times ranged from 45 to 180 min, in most cases with a sedation time of 60 min. Children aged 1 to 5 years accounted for 73.9% of the sedations evaluated, while children aged 10 to 12 years accounted for 3.8% of the population (Table 1).

### 3.2. Type of Dental Procedure

**Figure 1:**
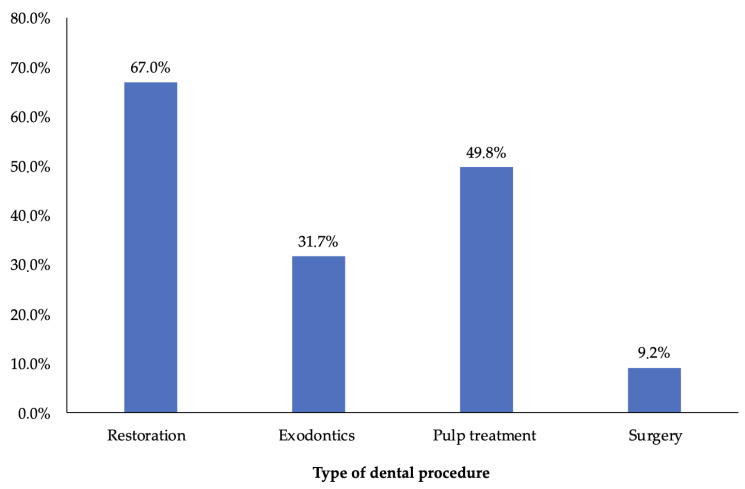
Type of dental procedure. Note: The most frequently performed procedures were restorations, followed by pulp treatment, followed by minor surgery.

The types of procedure performed per sedation session ranged from one to three: 49.1% of sedations were for a single type of dental procedure, 44.2% for more than one type of treatment, and 6.7% for three types of procedures. The main dental procedures performed were restorations (67%) and pulp treatment (49.8%) (Figure 1).

### 3.3. Number of Treatments According to Age

**Figure 2:**
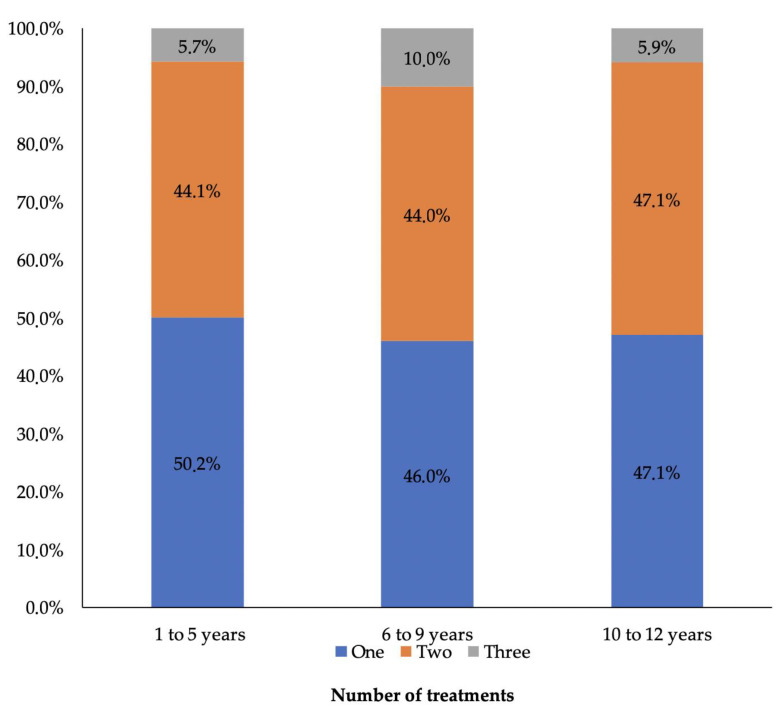
Number of dental treatments according to age group by sedation appointments. Note: In all groups, two types of procedures were generally performed; this table does not indicate the number of procedures.

Regarding the number of treatments performed according to age, it was established that 50% of children aged 1 to 5 years underwent sedation for only one type of dental procedure, while 10% of children aged 6 to 9 years underwent three types of treatment during the sedation process. No significant difference was reported between the number of treatments and the age group to which the children belonged (x = 2.42; *p* = 0.660) (Figure 2).

### 3.4. Type of Dental Procedure According to Age Group

**Figure 3:**
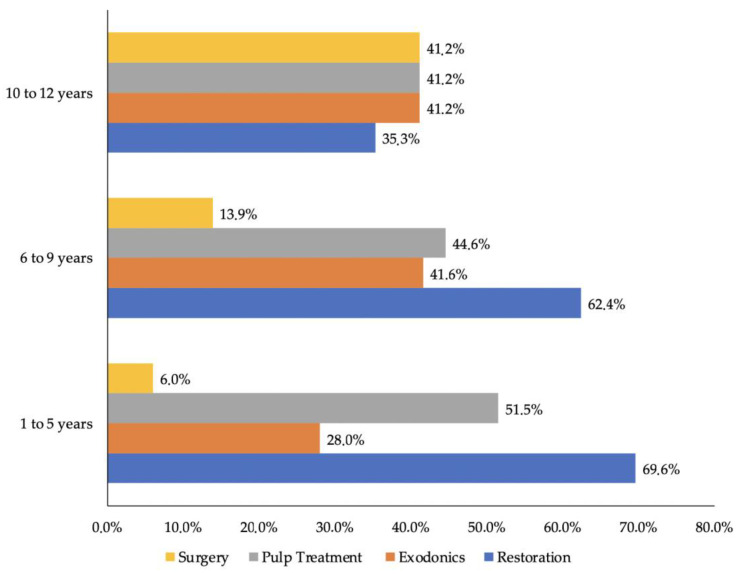
Type of dental procedure according to age group. Note: In the 1 to 5 year old and 6 to 9 year old groups, a higher percentage required sedation for restorations and pulp treatments.

It was determined that 69% of children from 1 to 5 years of age underwent restorations, representing the most frequent treatment performed of the procedures in this age group, followed by pulp treatment with 51.5%. In the 6 to 9 years of age group, it was determined that 62% underwent sedation for restorative purposes, followed by pulp treatments with a frequency of 44.6%. Finally, in the group of children aged 10 to 12 years, equal proportions (41.2%) underwent treatment for surgery, exodontia, and pulp treatment, showing a decrease in sedation for restorations. There was a significant difference in the performance of restoration between the age groups (x = 9.61; *p* = 0.08) as well as in exodontia (x = 7.59; *p* = 0.22) and surgery (x = 27.65; *p* = 0.000) (Figure 3).

### 3.5. Time According to Age and Number of Treatments

Finally, a descriptive analysis was performed on the types of treatments and the time allotted. It was determined that when having one or two types of dental procedures in any age group, the most common sedation time was 60 min, while when exposed to three types of procedures, the sedation time was 120 min. Sedation of up to 180 min was also recorded in children aged 1 to 10 years (Table 2).

## 4. Discussion

Dental caries is a common disease, affecting almost 100% of adults and between 60% and 90% of schoolchildren worldwide [7,24]. Sabbahi, D. [25] mentioned that dental caries in childhood are a major problem requiring treatment; several factors influence this situation, including inadequate management by parents, who wait for caries to reach an advanced stage with the presence of extensive and painful cavities. This leads to a significant problem at the pediatric level, as disease can cause pain and infection, which often can only be controlled by extensive restoration or extraction of the affected teeth. In addition to this, the psycho-emotional state of children should be considered, since an altered state leads to undesirable behaviors in the dental office, which jeopardize the results of the treatments, as well as the patients [11,13].

Historically, conventional protocols have been used for dental management at the pediatric level, such as the use of general anesthesia, which in many cases is not convenient owing to its possible adverse effects. Another drawback is the use of specialized medical equipment and facilities, which hinders its routine use [26]. Local anesthesia is also commonly used; however, some children may present dental fear or behavioral problems, complicating its use [27]. These situations tend to make dental management difficult, so it is necessary to apply an alternative to control anxiety and behavior. The application of sedation in a pediatric patient serves as a method to modify undesirable behavior and reduce the anxiety produced in the consultation [11,28].

Based on the needs for sedation in the case of Cuenca, Ecuador, it was found that the population analyzed was between 1 year 1 month and 12 years 9 months, with the highest percentage of sedation evaluated (73.9%) in children between 1 and 5 years, while children between 10 and 12 years only represented 3.8% of the population. This first result left us to conclude that since the majority of the sample requiring dental treatment under sedation was under 5 years of age, dental anxiety and/or behavior problems exist at very early ages, perhaps owing to age and their own behaviors. In addition, contact with a dentist may occur for the first time or the high incidence of dental caries, since when comparing the main treatments administered in this age range it was observed that restorations represented 69.6%. These values tended to be justifiable because reports have indicated that the prevalence of dental caries is greater than 80% in preschool children [29].

In recent decades, the prevalence and severity of dental caries in primary and permanent dentitions has been reduced in several countries according to published studies. However, despite this achievement, the prevalence and severity of dental caries remains high worldwide [30]. This can be seen in the case of Cuenca, Ecuador, where we observed that the most frequent treatment in the entire population was restorations at 67%, followed by pulp treatment at 49.8%, and exodontia at 31.7%, these last two clinical situations generally being the consequence of inadequate management of dental caries. Regarding the population aged 6 to 9 years, the values tended to be very similar to those of the first group in terms of restorations, indicating that within this age range the prevalence of caries was still high [31]. On the other hand, it could be seen that children between 10 and 12 years of age tended to require only 3.8% of the evaluated sedations, and at the same time the least frequent type of treatment performed was restorations, in comparison with children between 1 and 5 years of age. Children between 10 and 12 years of age presented a similarity in terms of the types of treatments performed, such as surgery, pulp treatment, and exodontia, which was an important observation, allowing us to differentiate the hygienic behaviors in terms of age. Since a lower percentage of restorations was observed, children in this age group appeared to present better oral care and hygiene by reducing the sedated population attended to in a dental office. It also allowed us to understand that dental behavior and anxiety are manageable in older children, permitting dental procedures to be performed without the need for sedation [12,17].

This study indicated that the need for sedation is high in preschool children and that it is clearly associated with dental caries, although the same situation was also observed in school-age children up to 9 years of age, and it became evident that after the age of 10 years this situation tended to decrease. Therefore, a question remained to be discussed in this study, and that was the question of how necessary sedation is at an early age, especially in dental restorations, since we can reduce its use with adequate measures of prevention and oral health, which would reduce the prevalence of dental caries. It should also be considered that pediatric dentists can lower stress levels when performing dental treatments [32], which is why they resort directly to the use of sedation, increasing the frequency of use. However, it is necessary for dentists to take measures that allow them to manage dental behavior and anxiety and not resort directly to sedation, since it requires specialized personnel, specialized equipment, and in some cases, patients may present adverse reactions [2,33]. In the case of sevoflurane, complications on awakening are rare, although some studies mention a significantly higher incidence of excitement/agitation, cough, and postoperative nausea and vomiting [34,35]. Among the most common adverse effects of remifentanil are apnea, muscle rigidity, nausea, vomiting, and hypotension [36]. Moreover, when propofol is given, the most common side effects include bradycardia, apnea, tachycardia, arrhythmia, hypertension, injection site pain, rash, and pruritus [37,38]. Finally, it must be considered that various animal studies suggest neurotoxicity as a potential long-term risk of anesthesia in young pediatric patients and newborns; however, to date there are no concrete clinical data to suggest that the use of anesthetics in newborns or young children is associated with signs of developmental neurotoxicity, for which concerns persist and continue to be a focus of research [38,39].

Among the limitations of the study, we can mention the lack of more detailed information on the procedures performed, the conditions of administration of the sedatives, and the most used drugs. However, at the level of Latin America and Ecuador, this is the first study that provides us with a starting point and seeks to encourage analysis and discussion of the importance and safety of outpatient sedation at the pediatric level in dentistry, since in the absence of regulations clear on dental sedation in each country of the region, there is no patient safety, this being a priority in health care. In the case of Ecuador, developing clear and specific regulations by the control entities would make it possible to regularize sedation services in the dental office for children and at the same time control possible adverse effects.

## 5. Conclusions

The need for sedation for dental procedures is high in preschool patients in the city of Cuenca, Ecuador. Ambulatory sedation has contributed to satisfy this need especially in situations where it is not possible to use operating rooms.

Professionals specialized in sedation must foresee the adverse effects after sedation, so the patients to be sedated must be carefully selected under well-defined clinical parameters, avoiding the possible neurological effects that are still under discussion. Given the high rates of pediatric dental pathologies, it is likely that the need for sedation will continue to grow soon. This situation will increase the success of sedation by refining the parameters of behavioral assessment using appropriate drugs and appropriate routes of administration.

It is essential that countries begin to develop regulations according to their reality, as in the case of Ecuador, that allow regulating ambulatory sedation for dental procedures and thus both health professionals and parents are well informed about the risks and benefits of sedation at the dental level.

## Figures and Tables

**Table 1:** Descriptive data on sedation according to age.

Age	Frequency		Sedation Time in Minutes		
	*n*	%	Maximum	Minimum	Mode
1 to 5 years old	331	73.9	180	45	60
6 to 9 years old	100	22.3	180	60	60
10 to 12 years old	17	3.8	120	60	60

**Table 2:** Time according to age and number of treatments.

Age	Number of Procedures	Treatment Time (Minutes)				
		Minimum	Maximum	Median	Mode	DE
1 to 5 years old	One	45	180	60	60	13
	Two	60	180	60	60	23
	Three	60	180	120	120	36
6 to 9 years old	One	60	120	60	60	12
	Two	60	180	60	60	29
	Three	60	180	120	120	49
10 to 12 years old	One	60	120	60	60	21
	Two	60	120	60	60	31
	Three	120	120	120	120	-

## Data Availability

https://ucacueedu-my.sharepoint.com/:f:/g/personal/epachecoq_ucacue_edu_ec/ErPSlyMaXgdPgnvgfrSTHTIBFJt01Wl9biegZJ87Iw23EQ?e=EBRHPa (accessed on 2 October 2022).
